# A Rare Cause of Nuchal Rigidity: Metastatic Triple-Negative Breast Cancer Involving the Skull and Upper Cervical Spine

**DOI:** 10.7759/cureus.74490

**Published:** 2024-11-26

**Authors:** Daniil Katkov

**Affiliations:** 1 Internal Medicine, Waterbury Hospital, Waterbury, USA

**Keywords:** metastatic triple-negative breast cancer, nuchal rigidity, photophobia, skull base metastases, spine metastases, triple-negative breast carcinoma

## Abstract

A 48-year-old female presented to the ED with worsening headache and neck pain for the past week. Her medical history is significant for recurrent left-sided triple-negative breast cancer (TNBC) with metastasis to the chest wall, liver, and lungs, initially diagnosed two years ago. She underwent a left-sided mastectomy and received radiation therapy and chemotherapy. Physical examination was remarkable for nuchal rigidity and photophobia. MRI of the brain with contrast was performed, and the results were consistent with the new calvarium, skull, and upper cervical spine osseous metastases without any intracranial metastatic disease. This case report presents a rare cause of nuchal rigidity and photophobia due to an uncommon metastatic pattern in a patient with TNBC.

## Introduction

Triple-negative breast cancer (TNBC) is an aggressive subtype of breast cancer that lacks the expression of estrogen receptors (ERs), progesterone receptors (PRs), and human epidermal growth factor receptor 2 (HER2) [1]. Accounting for approximately 10-20% of all breast cancers, TNBC is associated with a poorer prognosis compared to other breast cancer subtypes, primarily due to its higher likelihood of early metastasis and limited treatment options [2]. The absence of ER, PR, and HER2 precludes the use of targeted therapies, leaving systemic chemotherapy as the mainstay of treatment [3].

The metastatic pattern of TNBC often includes visceral organs such as the lungs, liver, and brain, as well as bones. However, metastasis to the skull and upper cervical spine is rare and can present unique clinical challenges [4,5]. This report presents a patient with metastatic TNBC who developed metastases in the skull and upper cervical spine, manifesting symptoms such as neck stiffness and light sensitivity, commonly seen in infectious or inflammatory diseases such as meningitis [6].

Nuchal rigidity and photophobia are unusual initial presentations for metastatic breast cancer. Advanced imaging techniques, such as MRI and PET scans, are critical in identifying and characterizing metastatic lesions in atypical locations [7].

## Case presentation

A 48-year-old female presented to the hospital with a worsening headache over the past week, accompanied by increased fatigue and altered mental status. She described the headache as dull, severe, and bilateral, with associated neck pain. The symptoms had been intermittent for the past six weeks, initially moderate but significantly worsening in the past six to seven days, accompanied by increased sensitivity to light. Due to these symptoms, she had become more bedridden. She denied any fever, chills, nausea, or vomiting.

Her medical history is notable for left-sided breast cancer, initially diagnosed two years ago. Biopsy findings were consistent with grade 3 triple-negative invasive ductal carcinoma. She underwent a lumpectomy and sentinel lymph node biopsy, which was negative, although lymphatic invasion was noted in the biopsy specimen. The tumor measured 12 mm in diameter, and an MRI of the brain at that time showed no signs of metastatic disease. She received two cycles of adjuvant chemotherapy with doxorubicin, cyclophosphamide, and paclitaxel but was lost to follow-up. Six months ago, a mammogram indicated local recurrence and further work-up revealed metastases to the chest wall, liver, and lungs. Biopsy confirmed invasive breast carcinoma with mixed ductal and lobular features, grade 3, ER-negative, PR-negative, and HER2-negative. Immunohistochemistry was positive for CK7, MOC31, and BerEP4 and negative for CK20 and GATA3. More recently, she was found to have a left-sided pleural effusion. Diagnostic thoracentesis was performed, and cytology analysis showed breast carcinoma cells with negative ER, negative PR, negative HER2, and high Ki-67. Immunohistochemistry was positive for MOC31 and negative for mammaglobin and GATA3. A special stain for mucicarmine was negative for intracytoplasmic mucin droplets. She received three weeks of carboplatin and paclitaxel but was again lost to follow-up. Besides breast cancer, her past medical history is remarkable for left upper extremity deep venous thrombosis, which was managed with low molecular weight heparin, chronic lower back pain, opioid use disorder, and anxiety disorder.

**Figure 1:**
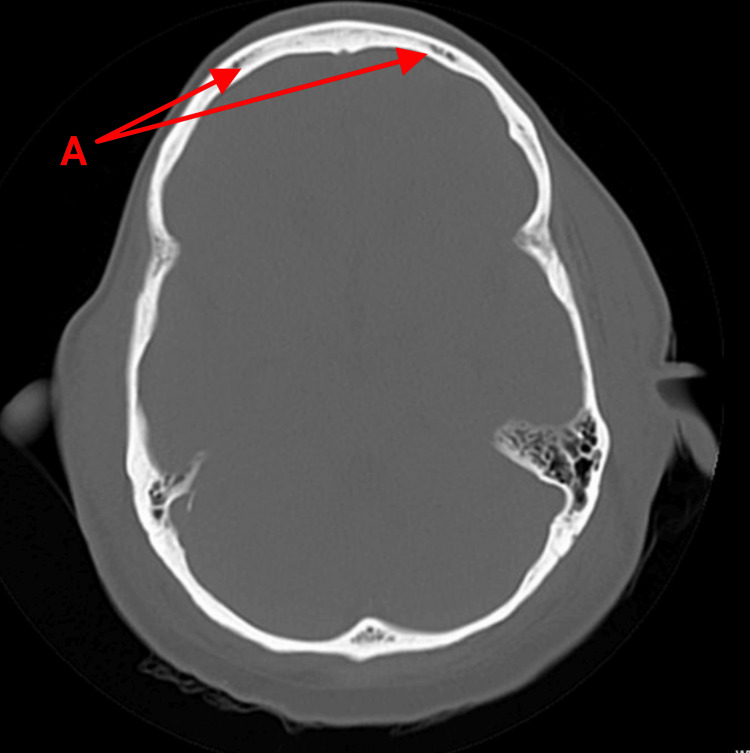
Computed tomography (CT) scan of the head A: The arrows originating from the letter "A" (highlighted in red) indicate patchy areas of the calvarium that are consistent with metastatic disease.

**Figure 2:**
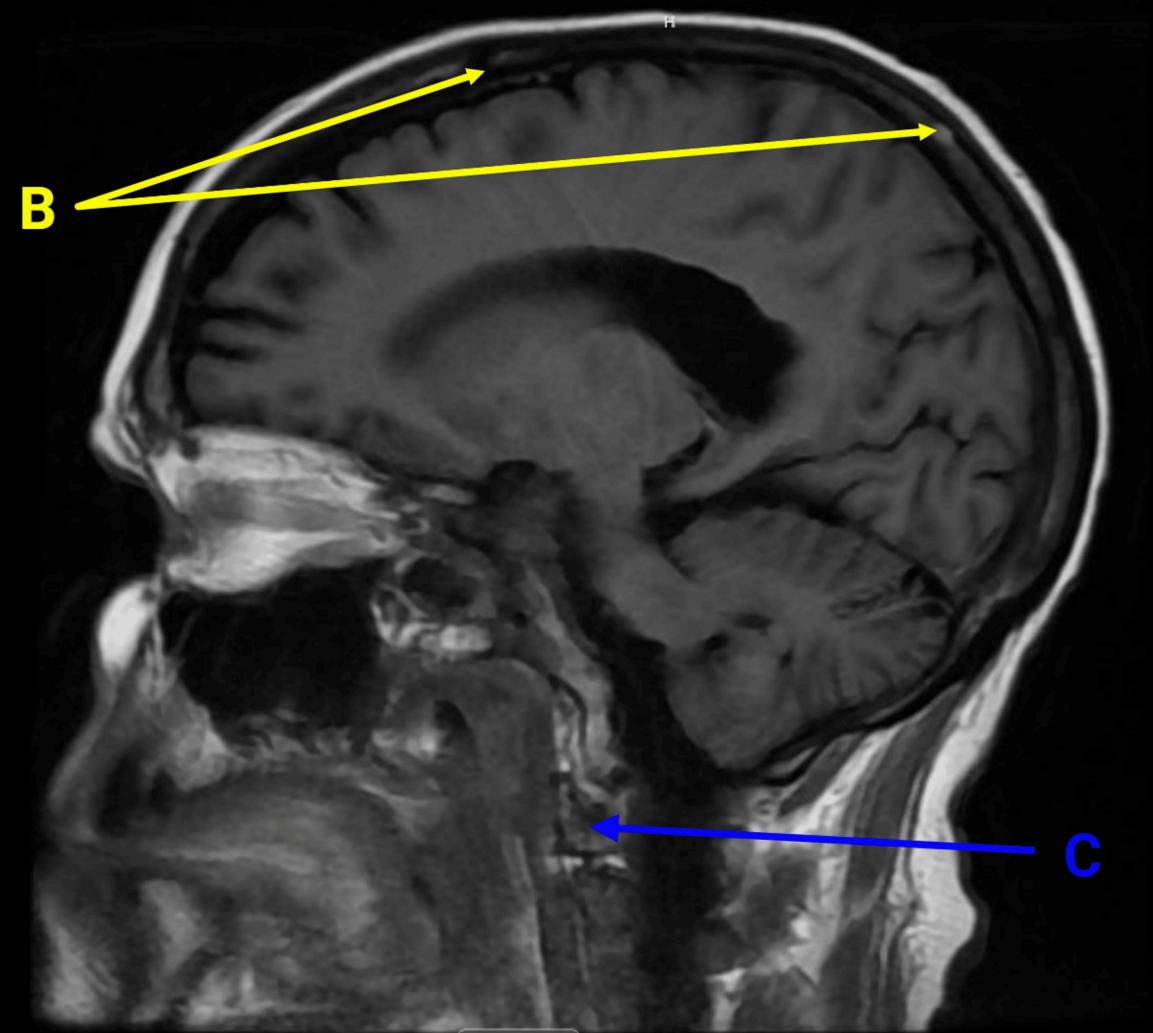
Magnetic resonance imaging (MRI) of the brain with intravenous contrast B: Patchy areas of abnormal T1 signal throughout the calvarium (highlighted in yellow), consistent with new osseous metastases. C: Similar patchy areas of abnormal T1 signal in the upper cervical spine (highlighted in blue), also indicative of osseous metastases.

On presentation, she was vitally stable and afebrile. Physical examination revealed nuchal rigidity but negative Kernig and Brudzinski signs. Increased sensitivity to light was also noted. Her CBC and basic metabolic panel were unremarkable. The peripheral smear did not show any bands, and she was negative for systemic inflammatory response syndrome. She had mildly elevated lactic acid. A non-contrast CT scan of the head showed no evidence of acute infarction, hemorrhage, or mass but revealed patchy areas of the calvarium concerning metastatic disease (Figure 1). MRI with contrast of the brain showed patchy areas of abnormal T1 signal throughout the calvarium, skull base, and upper cervical spine, consistent with new osseous metastases (Figure 2). No intracranial metastatic disease was identified, and no leptomeningeal enhancement was noted. The patient refused to undergo a lumbar puncture. Following further discussion, she was transitioned to inpatient hospice care and passed away after seven days.

## Discussion

Breast cancer is the most common malignant tumor worldwide, with about 70% of advanced breast cancer patients developing bone metastasis [8-10]. Interestingly, 60-75% of patients with metastatic breast cancer first present with bone metastases [11]. The spine is the most frequent site of bone metastasis in these patients [12]. Once it occurs, bone metastasis is usually difficult to manage and leads to significant bone-related complications, including pain, pathological fractures, hypercalcemia, and spinal cord compression. These complications severely reduce patients' quality of life and increase both mortality and morbidity [13].

Bone metastasis results from complex interactions between tumor cells and the bone microenvironment, aligning with Paget's "seed and soil" hypothesis [14]. Tumor cells undergo epithelial-mesenchymal transition to acquire invasive capabilities, degrade the extracellular matrix via fibrinolytic enzymes and matrix metalloproteinases, and migrate through the lymphatic and blood vessels. During circulation, cancer cells can form aggregates with platelets and leukocytes, increasing their resistance to shear stress and immune clearance. Additionally, the venous blood from the breast can bypass the pulmonary circulation through the vertebral venous system, explaining the propensity for axial bone metastasis [8].

The bone microenvironment, rich in blood supply and growth factors such as transforming growth factor-beta (TGF-β) and insulin-like growth factors, attracts and supports metastatic cells. These cells invade the bone marrow, establish their own blood supply, and secrete factors such as parathyroid hormone-related protein and ILs, promoting osteoclast differentiation and bone destruction. This leads to a vicious cycle of tumor-induced bone destruction, particularly in breast cancer, which often results in osteolytic lesions [8].

Metastatic cancer cells also modulate the immune system within the bone microenvironment. Tumor-derived factors such as vascular endothelial growth factor, TGF-β, and prostaglandin E2 recruit myeloid-derived suppressor cells that inhibit the tumoricidal activity of T lymphocytes, facilitating tumor cell survival [8,15].

TNBC exhibits a relatively distinct metastatic pattern compared to other breast cancer subtypes. While it tends to metastasize more frequently and earlier to visceral organs such as the lungs, liver, and brain rather than bones, which are more common sites for hormone receptor-positive or HER2-positive breast cancers, rare presentations such as metastasis to the skull and cervical spine have been observed. Studies indicate that 84% of women with TNBC presented with visceral metastases at the time of first metastatic spread compared to 61% for other breast cancer subtypes [5,16,17].

This atypical presentation of TNBC metastasis to the skull and cervical spine may be due to underlying biological differences. TNBC is characterized by aggressive behavior and rapid growth, often lacking the hormone-driven pathways that are more commonly seen in hormone receptor-positive cancers. This may explain why TNBC is more prone to visceral involvement early on and, in some cases, atypical sites such as the skull. On the other hand, hormone receptor-positive and HER2-positive breast cancers, which are typically slower growing and have bone tropism, tend to metastasize to bones more frequently than TNBC. Understanding these patterns can help clinicians anticipate and tailor surveillance and treatment strategies for patients with TNBC [5,7,8,12].

In this case report, the patient was found to have TNBC with metastases to the skull and upper cervical spine. Skull metastasis in TNBC is relatively uncommon compared to other metastatic sites. While brain metastases, including those involving the skull, occur in about 25% of women with metastatic TNBC, specific data focusing solely on skull metastases are limited [17,18].

The spine is a common site for bone metastasis, with the thoracic spine being the most frequently involved (60-70%), followed by the lumbosacral spine (20-25%), and then the cervical spine. Upper cervical spine metastases are rare, accounting for less than 1% of all spinal metastases. The prognosis for metastatic bone disease, particularly in cases of TNBC, is generally poor, with median survival often under a year due to the aggressive nature of TNBC and its limited treatment responsiveness compared to other breast cancer subtypes [19].

Treatment options for spinal metastases include radiation therapy, which is frequently employed to alleviate pain and control tumor growth, as well as bisphosphonates or denosumab to strengthen bones and reduce the risk of skeletal-related events. In selected cases, systemic therapies such as chemotherapy or targeted therapies (e.g., poly [ADP-ribose] polymerase inhibitors in BRCA-mutated TNBC) may also be considered, depending on the patient's overall condition and tumor biology. For this patient, a multidisciplinary approach involving radiation therapy and bone-modifying agents was carefully considered to manage both symptoms and disease progression while balancing the overall treatment burden before the patient was transitioned to hospice [10,17,18].

The patient's symptoms of severe head and neck pain could be explained by the metastatic spread of breast cancer. Nuchal rigidity could potentially be explained by direct invasion of the metastatic cancer with compression of underlying structures, including nerves and neck extension muscles. Spasm-related pain and the local effects of the tumor are also factors. Photophobia was most likely a nonspecific finding in this case and was related to metastatic spread and systemic illness due to breast cancer [6]. Another potential explanation is undiagnosed leptomeningeal carcinomatosis. A lumbar puncture was not performed due to the patient's refusal, and CSF was not analyzed in this case. Intracranial metastatic disease, particularly leptomeningeal carcinomatosis (LC), can be missed on MRI with contrast in certain cases. The sensitivity of MRI with contrast for detecting LC ranges from 75% to 90% [20]. Another potential differential diagnosis is subacute meningitis (e.g., tuberculosis, fungal); however, the patient did not have any specific exposures or other signs of infection, so this appears to be less likely. One more theoretical differential diagnosis for the patient's symptoms is opioid withdrawal, as the patient's poor oral intake could have led to an abrupt reduction in opioid use. Opioid withdrawal typically presents with symptoms such as agitation, anxiety, GI distress (nausea, vomiting, diarrhea), muscle aches, and nonspecific autonomic signs. However, opioid withdrawal alone would not account for the imaging findings observed in this case and does not fully explain the patient's clinical presentation.

## Conclusions

This case highlights the rare occurrence of skull and upper cervical spine metastases from TNBC, presenting with atypical symptoms of nuchal rigidity and photophobia. The patient's clinical presentation mimicked infectious or inflammatory conditions, such as meningitis, underscoring the importance of considering metastatic disease in the differential diagnosis for patients with known malignancies and unusual symptomatology. Advanced imaging techniques, such as MRI, were crucial in identifying the metastatic lesions and differentiating them from intracranial metastatic disease or leptomeningeal involvement. The aggressive nature of TNBC and its propensity for early and widespread metastasis, even to uncommon sites such as the skull and cervical spine, highlights the need for vigilant monitoring in these patients.

Given the rarity of skull and cervical spine metastases in TNBC, further research is essential to understand the mechanisms underlying this metastatic pattern. Investigating targeted management strategies specifically for metastases to these sites could offer potential therapeutic advances. Encouraging studies that explore new approaches, such as novel systemic therapies or localized treatment options, may improve clinical outcomes and quality of life for patients with metastatic TNBC. This case underscores the importance of early detection and tailored care plans while emphasizing the need for ongoing research into these uncommon metastatic presentations.
